# Beneficial Effects of Amnion-Chorion Stem Cell Grafting in the Long Term Management of Nonuremic Calciphylaxis Wounds

**DOI:** 10.7759/cureus.12170

**Published:** 2020-12-19

**Authors:** Theja Bhamidipati, Huy L Doan, Nariman Hossein-Javaheri, Hao T Tang, Mohsin Soliman

**Affiliations:** 1 General Surgery, Menorah Medical Center/Kansas City University, Overland Park, USA; 2 General Surgery, Kansas City University School of Medicine, Kansas City, USA; 3 General and Bariatric Surgery, Menorah Medical Center, Overland Park, USA

**Keywords:** advanced wound care, non uremic calciphylaxis, wound debridement, amnionic stem cell graft

## Abstract

Calciphylaxis is a poorly understood disease with high morbidity and mortality. The current primary literature on treatment is lacking; however, disease management often involves a multifaceted approach with a primary focus on consistent wound care. This report describes a case outlining the long-term management of nonuremic calciphylaxis wounds in a patient with severe malnutrition with the use of human amniotic membrane grafts, aggressive surgical debridement, nutritional therapy, and advanced wound healing techniques. A 38-year-old African American female with a history of non-uremic calciphylaxis presented from a transitional facility with numerous non-healing wounds in the setting of severe malnutrition secondary to bariatric surgery. Biweekly wound debridement was initiated utilizing an amniotic stem cell skin graft, dry applicable absorbent dressing, high-frequency ultrasonic ablation, and wound vacuum-assisted closure (VAC) over the course of approximately nine months. Nutritional supplementation was given in the form of jejunostomy tube feed due to a gastric bypass and a perforated viscus. At the current date, the patient demonstrates significant improvement in pain and wound healing. The patient is also able to ambulate with care and has begun steps towards independent management of wounds. Future goals of care include independent bedside wound management, placement of allograft, and discharge to a long-term care facility. Most patients with refractory pain, widespread necrotic wounds, and dangerous comorbidities will inevitably be referred to palliative care. This case creates a framework for the long term management of medically complex patients with nonuremic calciphylaxis using human amniotic membrane stem cell grafts and appropriate advanced wound care techniques.

## Introduction

Calciphylaxis is a rare disease with an incidence of 1%, but an increasingly prevalent disease characterized by pain, skin necrosis, non-healing ulcers, and potentially sepsis and death, with reported mortality rates often exceeding 50% [1, 2]. This devastating disorder has been most commonly described in patients with end-stage renal disease, hence its other name of calcific uremic arteriolopathy (CUA) [3]. However, In more recent literature, calciphylaxis in patients with an absence of renal disease termed nonuremic calciphylaxis (NUC), has become increasingly reported [4, 5]. Studies have also suggested numerous other risk factors for the development of calciphylaxis, including female gender, higher BMI, high levels of serum phosphorus, calcium, hypoalbuminemia, malnutrition, malignancy, inflammatory and autoimmune disease, diabetes, and warfarin use [6-8].

The characteristic skin lesions in calciphylaxis appear as necrotic ulcerations secondary to ischemia. Medial calcific sclerosis of the small arterioles in the dermis and superficial subcutis result in thrombotic occlusion and subsequent fibrosis [9]. The underlying pathogenesis of the calcifications, nonetheless, remains poorly understood. Hormonal disturbances, as well as oxidative stress from metabolic disorders, have been implicated in many forms of vascular calcification. The association of kidney disease and warfarin use with calciphylaxis indicates a possible relationship between excess calcium-phosphate products or decreased inhibitors of mineralization to the deposition of the amorphous calcium in tissue [10]. This, however, fails to explain the growing number of reported nonuremic calciphylaxis cases suggesting that additional mechanisms must be in play.

As with pathophysiology, a definitive guide for the management of calciphylaxis remains similarly unclear. Management is usually multidisciplinary, with therapies often targeting wound care and pain. Surgical debridement is one part of the regimen that has been associated with increased survival in patients with calciphylaxis [11]. Other techniques that are often implemented in treating chronic wounds, such as calciphylaxis, include negative pressure wound therapy, systemic antibiotics, proper dressings, and topical agents. Off-label trials of sodium thiosulfate (STS), with its antioxidant, antithrombotic, and anti-inflammatory properties, have also been frequently implemented in calciphylaxis treatment with uncertain efficacy [12].

The human amniotic membrane has historically been used as a biological dressing in the treatment of burns, diabetic ulcers, and various other insults of the integumentary system [13]. With increasing ease in preparation, stabilization, and storage, the use of human amniotic membrane grafts has become even more prevalent in the treatment of non-healing wounds that are otherwise refractory to advanced wound healing techniques [14]. Along with its anti-inflammatory and anti-microbial properties, the numerous growth factors and regulatory proteins present within the human amniotic membrane also help recruit mesenchymal stem cells, thereby increasing cellular proliferation, differentiation, and epithelialization while at the same time reducing fibrosis, which is beneficial wound healing [15]. With a clearly established place in the wound management repertoire, human amniotic membrane grafts may be a useful tool in treating calciphylaxis. 

Proper nutrition has also been a long-standing pillar of optimal and effective wound management. Protein malnutrition has been known to increase post-surgical infection rates by reducing innate and cell-mediated immune processes [16]. This can be particularly devastating in a patient with calciphylaxis, in which sepsis is the most common cause of death [1].

In this case report, we describe the use of human amniotic membrane grafts (AmnioFill®; MiMedx, Marietta, GA) in conjunction with frequent surgical debridement and advanced wound care techniques, including wound vacuum-assisted closure (VAC) therapy, to promote wound healing and reduce pain in a patient with nonuremic calciphylaxis. Moreover, recognizing the importance of nutrition within the paradigm of wound management, we also highlight the role of nutritional supplementation in this patient whose enteric absorption capability was severely limited.

## Case presentation

A 38-year-old African-American female patient was transferred from a long-term acute care facility with a chief complaint of non-healing wounds on the sacral region, bilateral thighs and flanks, and lower epigastrium. Upon arrival, the patient was placed in the intensive care unit (ICU) for electrolyte derangements, malnutrition, anemia of chronic disease, and sepsis as a result of necrotic development of multiple, bilateral, intra-pannus wounds. As per standard of care, trial treatment with sodium thiosulfate (STS) was immediately initiated.

**Figure 1:**
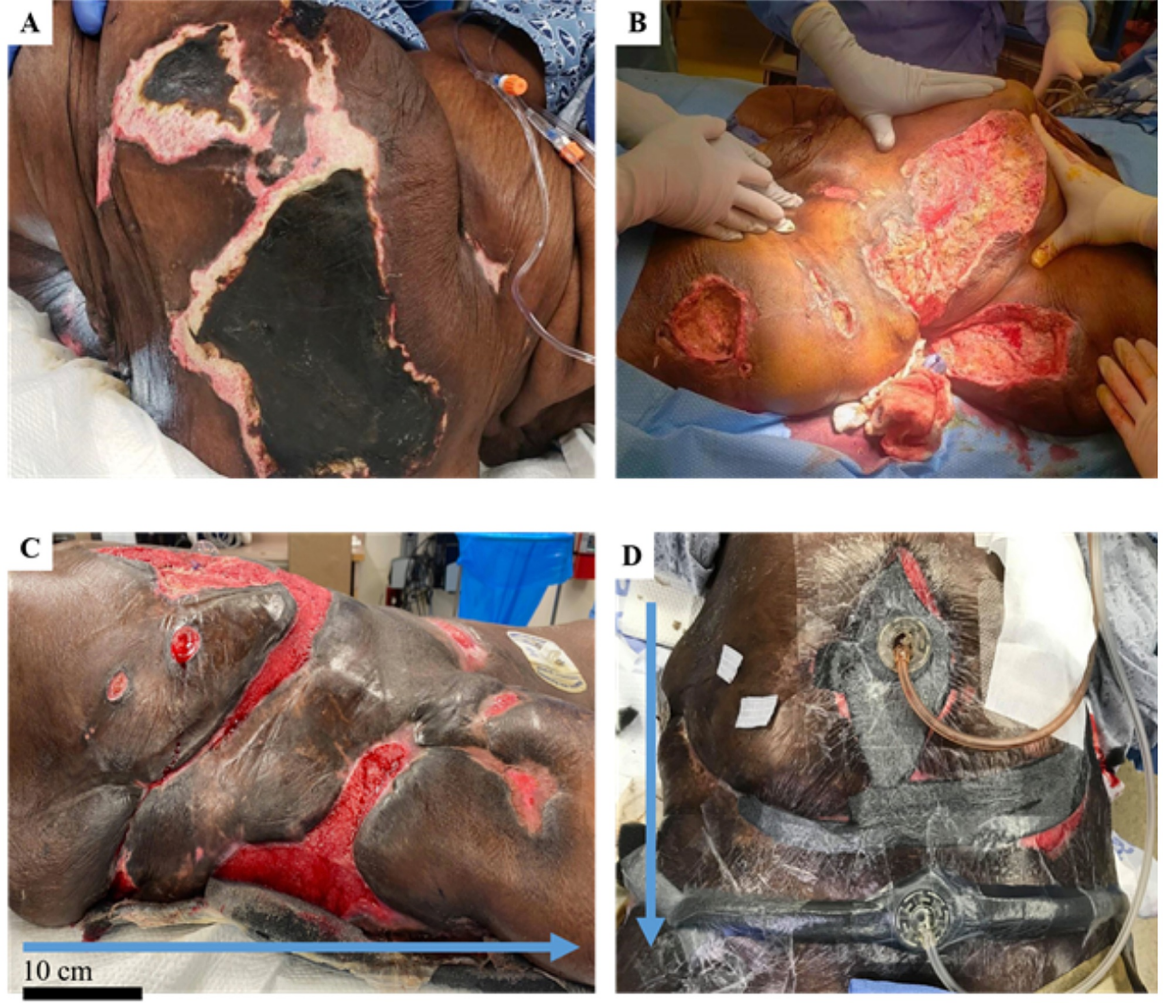
The initial presentation and improvement of wounds during the course of treatment A) Ulcerated lesions demonstrated as black eschar were the initial presentation of wounds located on the buttocks, flanks, hips, thighs, and panniculus. B) Cleared necrotic wounds following the utilization of the Misonix ultrasonic debridement device. C) Gradual improvement of the initial calciphylaxis wounds in addition to developed epigastric ulcer due to complications from bowel resection procedure. D) Wound vacuum-assisted closure (VAC) and packing to accelerate healing. The blue arrows indicate a cranial to caudal orientation. The scale bar indicates an approximate 10cm scale.

Biweekly surgical debridement was utilized to remove necrotic tissue and biofilm from wounds. Following endotracheal anesthesia and proper positioning, a combination of electrocautery and SonicOne® O.R. (Misonix, NY, USA), an ultrasonic debridement device was utilized to circumferentially remove slough, poorly formed granulation tissue or areas with potential keloid scars and nonviable skin/subcutaneous tissue along with muscle and fascia from all wounds (Figure 1A-B). Hemostasis was assured with electrocautery and direct pressure. The wounds were then packed with sterile saline-soaked Kerlix™ gauze (Vitality Medical, UT, USA) and dressed with Medipore™ tape (3M Corporate, MN, USA). An amniogel amniotic stem cell graft with growth factors was used to promote the healing of deep non-healing wounds at the epigastric region (Biohealing, Paruba, Czech Republic). The wound care team then reapplied Silverlon dressing (Silveron, IL, USA) and overlying VeraFlo™ wound VACs (3M Corporate, MN, USA) to the entirety of all wounds. Finally, Mepilex® wound dressing (Mölnlycke Health Care, GA, USA) was applied to well-heeled areas to prevent infection (Figure 1C).

**Figure 2:**
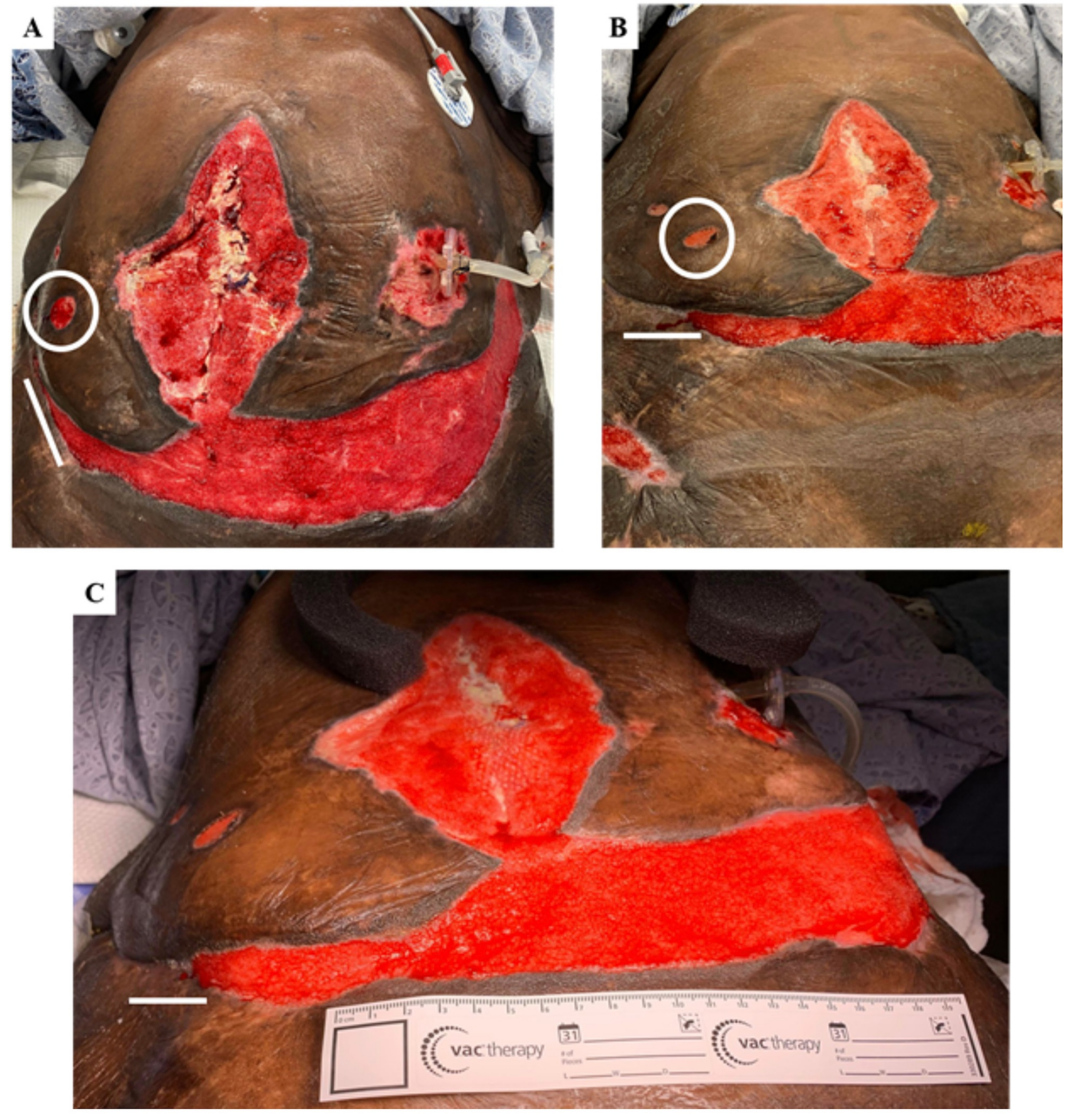
Treatment improved morphology Depicted A-C are anterior abdominal and pannicular wounds. White circles are referring to a minor wound at the abdominal right-lower-quadrant and the white lines are indicative of the right-pannicular wound.

Frequent complications occurred during the patient’s hospitalization, including recurrent sepsis, frequent watery diarrhea, acute respiratory failure, and perforated ileum resulting in small bowel resection. The abdominal surgical closure also dehisced and progressed to a large necrotic wound on the anterior abdomen/epigastric region (Figure 2). The patient’s healing process was complicated due to protein-calorie malnutrition secondary to a previous surgical history of roux-en-y gastric bypass and aggressive post-bariatric weight loss in addition to her calciphylaxis. In order to accelerate her recovery, her caloric deficiency was targeted utilizing total parenteral nutrition (TPN), tube fed supplementation, and regular diet per os (PO). Following treatment, her nutritional status steadily improved, as seen in Table 1. Despite her complicated medical history, continuous monitoring of her renal function, including blood urea nitrogen (BUN), creatinine, and glomerular filtration rate (GFR), indicated that she was not suffering from any kidney disorder and her calciphylaxis was therefore nonuremic in nature.

**Table 1:** Patient's laboratory and nutritional values prior to and after initiation of treatment for caloric deprivation BUN - blood urea nitrogen; GFR - glomerular filtration rate

Variables	Initial results	Six months post-treatment	One year post-treatment	Reference range
BUN (mg/dl)	21	15	18	6-22
Creatinine (mg/dl)	0.9	0.6	0.6	0.5-1.3
GFR (ml/min)	89.9	143	143	>60
Random glucose (mg/dl)	79	98	89	70-99
Calcium (mg/dl)	7.5	8.5	10.1	8.5-10.1
Total protein (gm/dl)	5.2	6.6	7.1	6.5-8.2
Albumin (gm/dl)	1.9	1.1	2.3	3.5-4.8

Surgical debridements were performed consistently over the course of approximately one year as per the patient’s tolerance to the procedure with particular attention to the following areas: left hip, bilateral thighs, bilateral panniculi, right flank, and sacral decubitus wounds. The abdominal lesion was primarily vacuumed and not subjected to continuous debridement. This treatment significantly improved her wounds on gross examination (Figure2). Although opioids did not improve the patient's wound healing, her pain was controlled by opioid polypharmacy as managed by a dedicated pain team.

**Figure 3:**
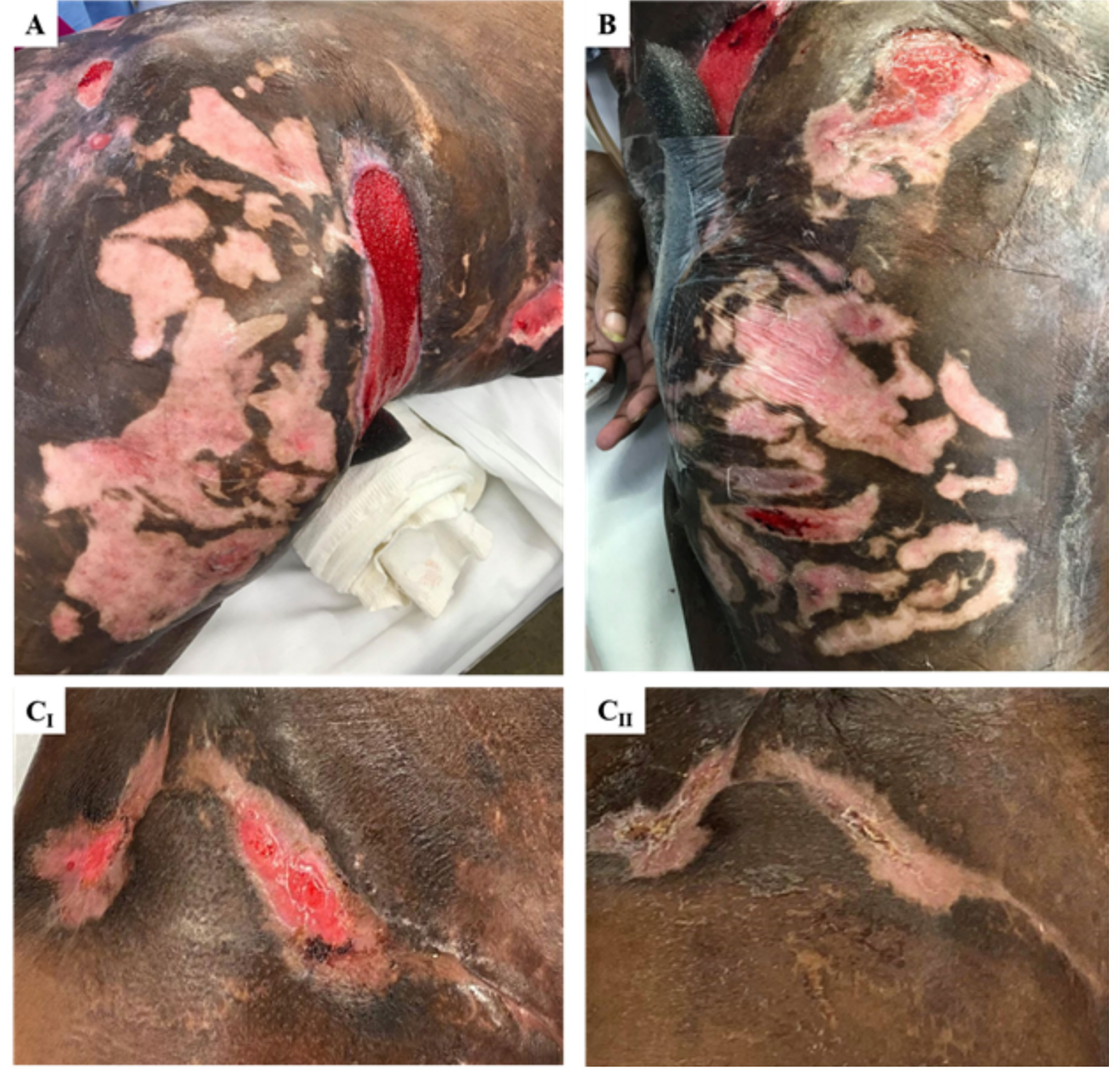
Calciphylaxis wounds healed by secondary intention The clear proliferation of granulation tissue in the right posterolateral (A) and left posterolateral (B) thighs. The right anteromedial thigh is represented within two months of treatment (progressive maturation from CI to CII). Images were obtained prior to covering the affected tissue with DuoDERM; however, when reached the level of CII, wounds were not covered at all as they were deemed healed.

Posterior wounds recovered more rapidly in comparison to the anterior lesions, and the proliferation and maturation of granulation tissue were evident (Figure 3). Due to their delicacy, wounds containing granulation tissue were not vacuum sealed but covered by DuoDERM® (ConvaTec, OK, USA) instead. This approach provided a hydrous wound-healing environment for the newly formed tissue and increased rates of epithelialization. No evidence of eschar was observed following treatment, and as of date, the patient has not developed any novel complications.

## Discussion

This patient's initial presentation with NUC and severe wound necrosis was complicated by malnutrition and leukocytosis offered a bleak outlook on survival. Palliative care was consulted during the early weeks of the intensive care unit (ICU) admission due to the low three-month survival. This report explores how early intervention with amnion/chorion stem cell graft and a comprehensive nutritional plan allowed the patient to make significant strides toward recovery with an increase in independence and eventual plan of allograft skin implantation and discharge to the long term care facility. 

The first highlight of this case is the use of a novel micronized amniotic/chorionic membrane (AmnioFill, MiMedx, Marietta) prepared as a skin biofilm on the deeper necrotic wounds across the patient pannus, chest, and sacrum. After administration of this biofilm, there was a noticeable improvement in the healing of the deeper wounds of the abdomen and chest in approximately one month (as evidenced by wound progression in Figures 2 and 3C). This unique biofilm contains placental cells derived from the amniotic or chorionic membrane, which are privileged tissues with antimicrobial properties. These tissues also contain stem cells, which retain pluripotent and multipotent features. When compared to typical grafts used in wound care, such as allografts or xenografts, these chorion/amnion grafts provide a structural matrix that promotes cell migration as well as new cell maturation at the site of injury. Thus, the addition of biologic growth factors exert a more potent effect on amniotic stem cells, creating an extracellular matrix for healthy tissue to grow upon [17]. The posterior wounds were specifically treated with additional amniotic growth factors due to the risk of repeated decubitus ulcers. Other ulcers of lower grade were treated with conventional foam absorbent (Mepilex) dressing and vacuum closure, which was sufficient for infection prevention. 

Early clinical outcomes were complicated by the patient’s inability to absorb appropriate nutrients. This not only complicated the delicate homeostasis of phosphate and calcium, which has been directly linked in the pathogenesis of calciphylaxis [18] but also prevented proper wound healing and allowed for necrotic growth and slough to continue to build. This, in turn, led to a general protein/calorie malnutrition (albumin <2.0), which progressed to the aforementioned complications such as recurrent bacteremia, hypoxic respiratory failure, small bowel perforation, and wound dehiscence. However, the aggressive protein-rich and calorie-rich diet was still inadequate, given the patient’s history of gastric bypass surgery and duodenal switch. More so, concurrent depression and anxiety associated with prolonged hospital stay decreased the patient’s appetite. 

The use of jejunostomy feeding bypassed the effects of bariatric surgery by administering food bolus directly to the viable jejunum distal to the duodenum and allowed the patient to get the necessary nutrition and electrolytes. As evidenced by Table 1, the timing of this improved nutritional status marked a noticeable improvement in the patient’s clinical course, with large wounds beginning to show healthy scar tissue.

Despite this improvement in nutritional care, the patient's clinical course was not linear, with fallbacks and complications associated with nearly every body system. Catheter bacteremia, pneumonia, perforated abdominal viscera, depression, and additional pressure ulcers all added undue stress on the patient’s recovery. A multifaceted approach with interdisciplinary teams was required to handle these complications and ensure a healthy environment to promote maximal nutritional intake.

At the current date, the patient reports much more controlled pain, has increased mobility and is on a completely regular PO diet. Furthermore, surgical debridement is being replaced by gradual bedside wound care as per the patient’s pain tolerance. In the upcoming weeks, continuous consultation with physical therapy (PT) and occupational therapy​​​​​​​ (OT) will be integral to ensure a healthy transition out of the hospital to a long-term care facility. Lastly, as the wounds begin to heal evenly with constant margins and stable scar tissue, a skin allograft is planned to finally close the wounds.

## Conclusions

This case outlines the use of amnion/chorion stem cell graft in the long term management of calciphylaxis. More so, this case highlights the importance of proper nutritional maintenance in the treatment of chronic and non-healing wounds in a patient who developed calciphylaxis with malnutrition from previous bariatric surgery.
